# Resminostat induces changes in epithelial plasticity of hepatocellular carcinoma cells and sensitizes them to sorafenib-induced apoptosis

**DOI:** 10.18632/oncotarget.22775

**Published:** 2017-11-30

**Authors:** Jitka Soukupova, Esther Bertran, Irene Peñuelas-Haro, Uxue Urdiroz-Urricelqui, Matthias Borgman, Hella Kohlhof, Isabel Fabregat

**Affiliations:** ^1^ “TGF-β and cancer” group, Oncobell Program. Bellvitge Biomedical Research Institute, (IDIBELL), L´Hospitalet, Barcelona, Spain; ^2^ Department of Physiological Sciences, Faculty of Medicine and Health Sciences. University of Barcelona (UB), L´Hospitalet, Barcelona, Spain; ^3^ 4SC AG, Planegg-Martinsried, Germany; ^4^ Immunic AG, Planegg-Martinsried, Germany

**Keywords:** HDAC inhibitors, HCC, EMT, resminostat, sorafenib

## Abstract

Resminostat, a novel class I, IIb, and IV histone deacetylase inhibitor, was studied in advanced hepatocellular carcinoma (HCC) patients after relapse to sorafenib (SHELTER study). In this phase I/II clinical trial, combination of sorafenib and resminostat was safe and showed early signs of efficacy. However, the molecular mechanisms behind this synergism have not been explored yet. In this work, we aimed to analyze whether resminostat regulates epithelial-mesenchymal and stemness phenotype as a mechanism of sensitization to sorafenib. Three HCC cell lines with differences in their epithelial/mesenchymal characteristics were treated with resminostat and sorafenib alone, or in combination. Resminostat prevented growth and induced cell death in the HCC cells, in a time and dose dependent manner. A collaborative effect between resminostat and sorafenib was detected in the mesenchymal HCC cells, which were insensitive to sorafenib-induced apoptosis. Expression of mesenchymal-related genes was decreased in resminostat-treated HCC cells, concomitant with an increase in epithelial-related gene expression, organized tight junctions and reduced invasive growth. Moreover, resminostat down-regulated *CD44* expression, coincident with decreased capacity to form colonies at low cell density. Conclusion: Resminostat shifts mesenchymal cells towards a more epithelial phenotype, lower invasive and stemness properties, which may contribute to the sensitization to sorafenib-induced apoptosis.

## INTRODUCTION

Human hepatocellular carcinoma (HCC) is the second most common cause of cancer-related death in men worldwide with increasing incidence [1]. Currently, sorafenib, a multikinase inhibitor, is the only approved drug for patients with advanced HCC, providing survival advantage of 3-months over the non-treated group [2]. Primary or acquired resistance is frequent. Therefore, novel therapies are needed for patients with advanced HCC after the failure of sorafenib.

Recent evidences pointed out that apart from an accumulation of genetic alterations of tumor-suppressor genes and oncogenes, epigenetic processes play an important role in HCC development [3, 4]. Histone deacetylases (HDACs) modulate gene expression by enzymatic removal of acetyl groups from lysine residues in both histones and other proteins. 18 HDACs have been identified so far and classified into four groups: the class I HDACs (HDAC1, 2, 3 and 8), class IIa HDACs (HDAC4, 5, 7, 9), class IIb HDACs (HDAC6 and 10), class III HDACs (sirtuins) and class IV HDACs (HDAC11). Inhibitors of HDACs are attractive epigenetic modulating agents due to low toxicity and frequent upregulation of HDACs in cancers. Specifically in HCC, HDAC1 expression was reported to be directly correlated with aggressiveness and could be used as a prognostic factor in patients with HCC after surgery [5]. As a consequence, down-regulation of HDAC1 was suggested as a novel treatment strategy in HCC [6]. Importantly, HDAC1 downregulation by sh-RNA [6] or by a specific inhibitor [7] showed low toxicity to normal hepatocytes. Other HDACs were also found to be up-regulated in HCC, such as HDAC2 [8] or HDAC3 [9, 10].

Resminostat is a novel orally available HDAC inhibitor, inhibiting classes I, IIb and IV of HDACs. The efficacy of resminostat *in vitro* was first evaluated in multiple myeloma cells. Inhibition of HDACs 1,3 and 6 was demonstrated at nanomolar concentration and the efficacy to abrogate cell growth and to induce apoptosis at micromolar concentration [11]. Safety of resminostat and the recommended dose were evaluated in a phase I study in patients with advanced solid tumors [12] and confirmed in a group of Japanese patients [13]. The recently finished SHELTER study, a phase I/II clinical trial in patients no longer responding to sorafenib, compared the anti-tumor efficacy of resminostat alone or in combination with sorafenib. Safety of mono- and combination therapy was demonstrated and the combination therapy revealed an advantage in terms of overall survival and time to progression. Moreover, the transcription factor zinc finger protein 64 (ZFP64) was identified as a prognostic and putative predictive factor for overall survival [14]. In a follow-up study, a phase I/II clinical trial of sorafenib alone or in combination with resminostat as first line therapy in Asian patients did not reveal overall survival benefits of the combination treatment. However, stratification of patients, based on HBV, platelet counts or non-prior treatment showed favourable results [15].

The molecular mechanism that could explain the synergism between resminostat and sorafenib has not been explored yet. In previous studies, we described that lack of response to sorafenib in HCC cells correlates with a mesenchymal phenotype and the expression of the stem-related gene CD44 [16]. Therefore, here we performed an *in vitro* analysis of the effects of resminostat on the mesenchymal and stemness phenotype in HCC cells as a possible mechanism of sensitization to sorafenib-induced cell apoptosis.

## RESULTS

### HCC cells are sensitive to resminostat induced cell death

**Figure 1:**
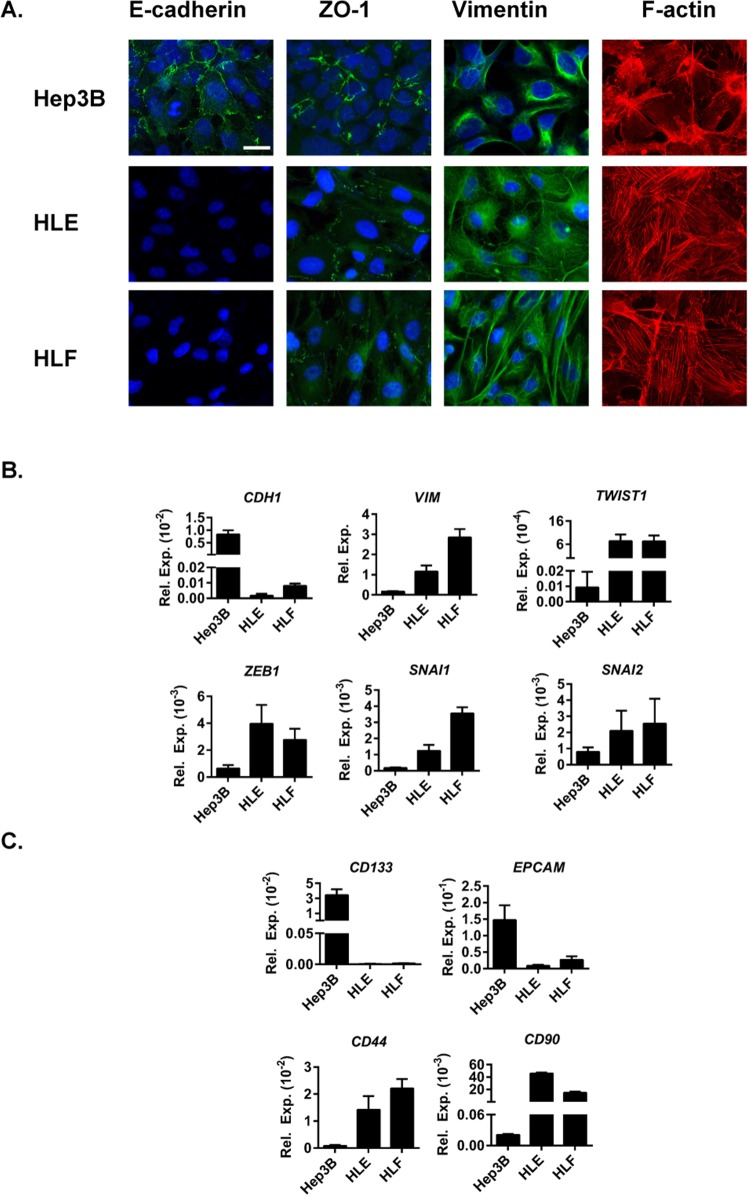
Characterization of HCC cell lines used in the study **(A)**. Immunofluorescence analysis of E-cadherin (green), ZO-1 (green), vimentin (green), F-actin (red) and DAPI (blue). Scale bar =25 μm. **(B+C)**. mRNA expression levels detected by qRT-PCR normalized to housekeeping gene *L32*. Mean±SD (n=3). **(B)**. EMT-related genes *CDH1, VIM* and EMT-inducing transcription factors *TWIST1, ZEB1, SNAI1 and SNAI2*. **(C)**. Stemnesss-related genes *CD133, EPCAM, CD44 and CD90*.

For this study we have selected three representative HCC cell lines with differences in their epithelial/mesenchymal phenotype. We have selected an epithelial Hep3B cell line, with high expression of an epithelial marker E-cadherin and a marker of tight junctions, Zonula occludens-1 (ZO-1), organised in cell membranes. On the other hand, two mesenchymal HCC cell lines were used: HLE and HLF, with high expression of a mesenchymal marker vimentin and a presence of stress fibres (F-actin) (Figure 1A). A mesenchymal phenotype, characterised by low expression of E-cadherin (*CDH1*), high expression of vimentin (*VIM*) and high expression of EMT-inducing transcription factors, such as *TWIST1, ZEB1, SNAI1* and *SNAI2* (Figure 1B) moreover correlates with the expression of cancer stem cell markers *CD44* and *CD90* (Figure 1C). Correspondingly, the epithelial phenotype with high *CDH1* expression, low expression of *VIM* and low expression of EMT-inducing transcription factors (Figure 1B) correlates with high expression of cancer stem cell markers *CD133* and *EPCAM* (Figure 1C).

**Figure 2:**
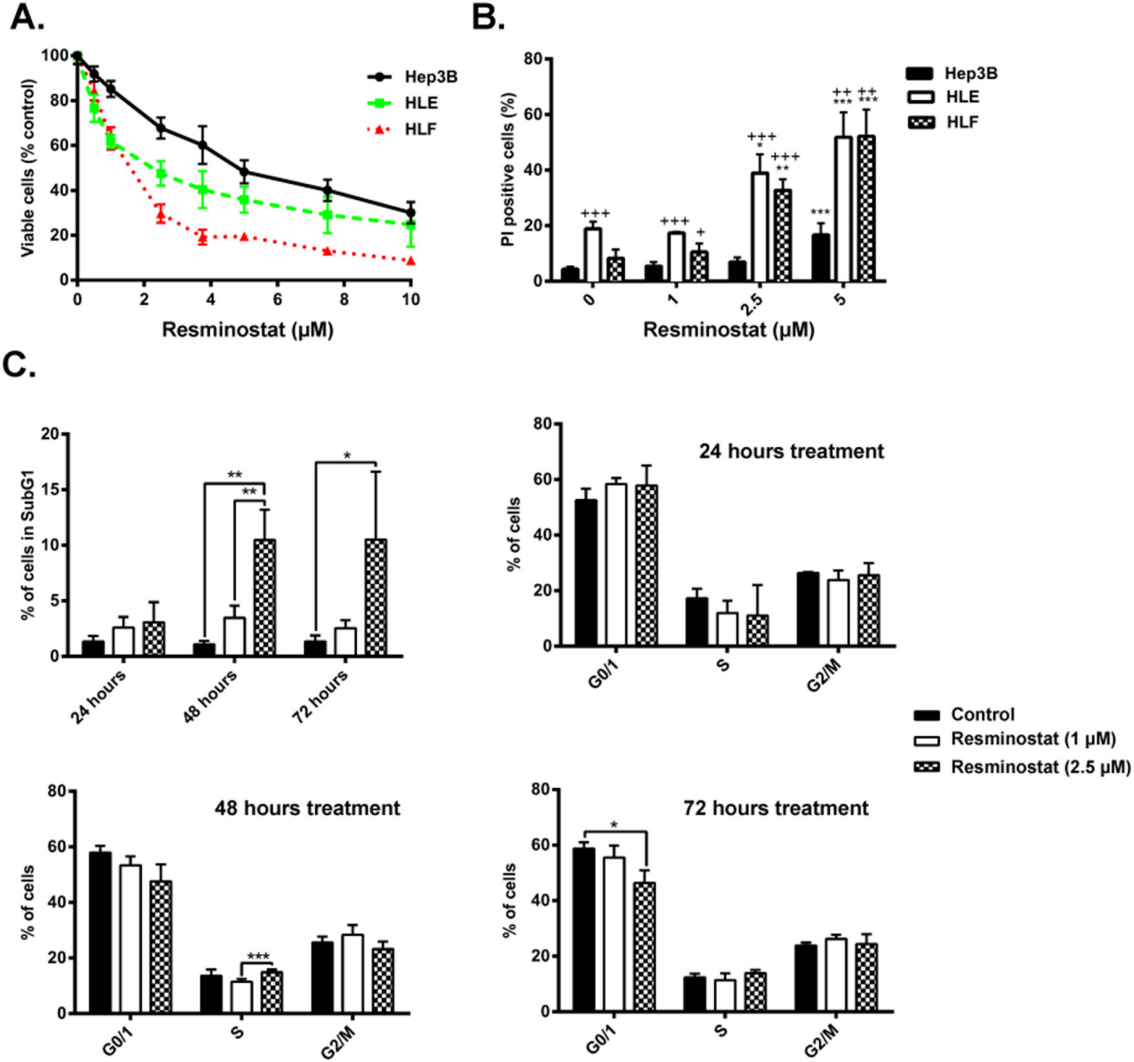
Response of HCC cells to resminostat treatment **(A)**. Hep3B, HLE and HLF were treated with resminostat (0-10 μM) for 72 h. Viable cells were analysed by crystal violet staining and normalised to control. Mean±SD (n=3). **(B)**. Hep3B, HLE and HLF were treated with resminostat (0, 1, 2.5, and 5 μM) for 72 h. The % of non-viable cells (PI-positive) was determined by flow cytometry. Mean±SD (n=3). ^*^ p< 0.05, ^**^ p<0.01, ^***^ p<0.001 as compared to control. + p<0.05, ++ p<0.01, +++ p<0.001 as compared to Hep3B. **(C)**. HLF cells were treated with resminostat (0, 1 and 2.5 μM) for 24, 48 and 72 h. 2×10^5^ cells of respective samples were fixed with ethanol, and stained using PI for cell cycle analysis. Mean±SD (n=3). ^*^ p< 0.05, ^**^ p<0.01, ^***^ p<0.001.

First, we analyzed the effect of resminostat (0-10 μM) on cell viability in those three HCC cell lines by crystal violet staining. The cell lines exhibited dose-dependent sensitivities to the cytotoxic effects of resminostat, presenting IC50 of 5.9, 3.7 and 2.0 μM (Hep3B, HLE and HLF, respectively: Figure 2A). The analysis of the induction of cell death by resminostat revealed higher percentage of dead cells (PI-positive) in resminostat-treated cells, being the mesenchymal HLE and HLF the more sensitive at lower resminostat dosage (2.5 μM) (Figure 2B and Supplementary Figure 1). Analysis of cell cycle after 24, 48 and 72 h of resminostat treatment (0-2.5 μM) in HLF cells presented a dose and time dependent induction of subG1 cells, with only modest changes in other phases of the cell cycle (Figure 2C). The induction of subG1 phase was also observed in resminostat-treated Hep3B and HLE cells (Supplementary Figure 2).

### Effect of combined treatment of resminostat and sorafenib in HCC cells

**Figure 3:**
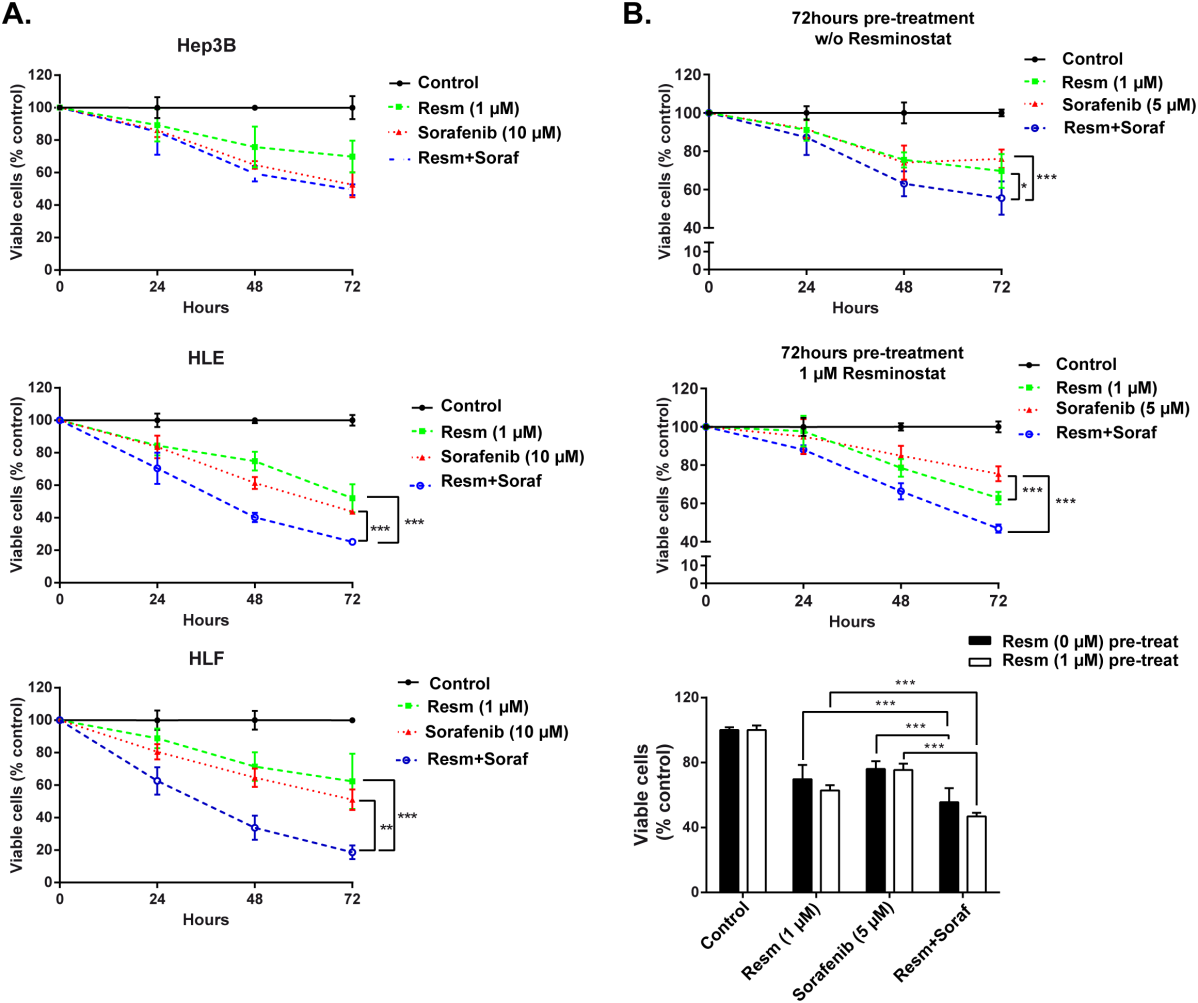
Pre-treatment with resminostat sensitizes mesenchymal HCC cells to sorafenib-induced apoptosis **(A)**. Hep3B, HLE, HLF cells were pre-treated without or with resminostat (1 μM) for 72 h. Cells pre-treated without resminostat were later treated without (Control) or with sorafenib (10 μM). Cells pre-treated with resminostat (1 μM) were later treated with resminostat (1 μM) or a combination of resminostat (1 μM) and sorafenib (10 μM). Viable cells were analyzed by crystal violet staining after 24, 48 and 72 h of respective treatments and normalized to control (resminostat 0 μM). Mean±SD (n=3). ^**^ p<0.01, ^***^ p<0.001. **(B)**. HLF cells were pre-treated in the absence (top graph) or in the presence of resminostat 1 μM (middle graph) for 72 h and later treated without (Control) or with resminostat (1 μM), sorafenib (5 μM) or a combination of resminostat (1 μM) and sorafenib (5 μM). Viable cells were analyzed by crystal violet staining after 24, 48 and 72 h of respective treatments and normalized to control (resminostat 0 μM). A comparison between the pre-treatment in the absence (black bars) and in the presence of resminostat (1 μM, white bars) is shown in the bottom graph. Mean±SD (n=3). ^***^ p<0.001.

To examine the effect of the combined treatment on cell viability, Hep3B, HLE and HLF cells were treated with resminostat at low toxicity dose (1 μM) for 72 h. Later, cells that were not pre-treated with resminostat were maintained under control conditions or treated with sorafenib. Cells pre-treated with resminostat (1 μM) continued to be treated with resminostat (1 μM) or with a combination of resminostat (1 μM) and sorafenib (10 μM) for up to 72 h and cell viability was determined by crystal violet staining. Our results revealed that in the mesenchymal HLE and HLF cells resminostat treatment enhanced sorafenib-induced decrease in cell viability, in a time-dependent manner (Figure 3A). More detailed analysis in HLF cells revealed that resminostat pre-treatment was not necessary in order to observe the collaborative effect. Furthermore, the synergistic effect was observed even at lower sorafenib concentration (5 μM) (Figure 3B). Considering this result, all the following experiments were performed at these concentrations and after simultaneous addition of both drugs.

**Figure 4:**
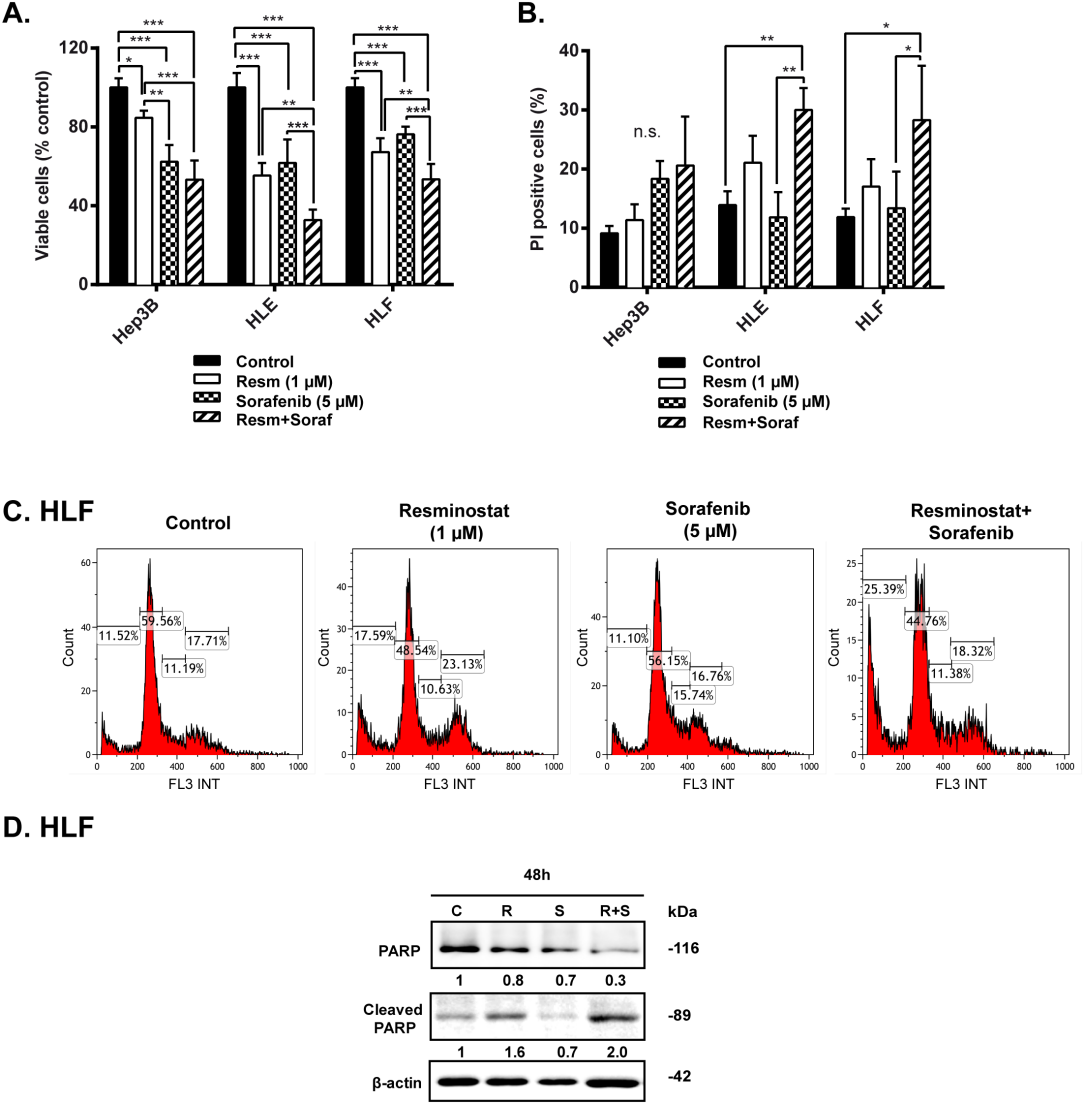
Effect of combined treatment with resminostat and sorafenib in HCC cells **(A+B)**. Hep3B, HLE and HLF cells were treated in the absence (Control) or in the presence of resminostat (1 μM), sorafenib (5 μM) or a combination of resminostat (1 μM) and sorafenib (5 μM) for 72 h. **(A)**. Viable cells were analyzed by crystal violet staining after 72 h of respective treatments and normalized to control (resminostat 0 μM). Mean±SD (n=3). ^*^ p< 0.05, ^**^ p<0.01, ^***^ p<0.001. **(B)**. The % of non-viable cells (PI-positive) was determined by flow cytometry. Mean±SD (n=3). ^*^ p< 0.05, ^**^ p<0.01. **(C)**. Representative images of cell cycle in HLF cells after combinatory treatment. **(D)**. Western blot analysis of PARP and cleaved PARP in HLF cells after 48 h of respective treatments. β-actin was used as a loading control. Densitometry analysis is below respective bands and normalized to β-actin.

To know whether the IC50 or resminostat changed in combination with sorafenib, we treated the Hep3B, HLE and HLF cells with sorafenib (5 μM) and resminostat in a range of concentrations (0-10 μM) (Supplementary Figure 3). Worthy to note that the calculated IC50 of resminostat slightly decreased in combination with sorafenib (5 μM), being 4.9 μM for Hep3B, 2.1 μM for HLE and 1.5 μM for HLF cells. Next, we maintained Hep3B, HLE and HLF cells under control conditions (resminostat 0 μM) or treated with resminostat (1 μM), sorafenib (5 μM) or a combination of both and analyzed cell viability by crystal violet staining (Figure 4A). Interestingly, in HLE and HLF cells, where sorafenib arrested proliferation but did not induce loss of cell number (Supplementary Figure 4), the combination of resminostat and sorafenib induced a significant enhancement of cell death (Figure 4B and Supplementary Figure 5). The analysis of cell cycle in HLF cells demonstrated an induction of subG1 phase (characteristic of apoptosis) with only modest changes in other phases of the cell cycle (Figure 4C). Furthermore, analysis of the cleavage of poly ADP ribose polymerase (PARP, target of caspase-3 during the apoptosis process), was higher in HLF cells treated with the combination of resminostat and sorafenib, as compared to resminostat treatment (Figure 4D). The cleaved form of PARP was barely detected after sorafenib treatment, which reinforced the lack of apoptosis response of these cells to sorafenib. These effects correlated with collaborative effects of resminostat and sorafenib in decreasing levels of pERKs, one of the well-known sorafenib targets (Supplementary Figure 6). At short time of sorafenib treatment, cells showed activated ERKs phosphorylation, as a mechanism of defence against the drug. In the presence of resminostat, this activation was abolished. Furthermore, at longer times, when sorafenib inhibits ERKs phosphorylation, the presence of resminostat amplified the effect. HDAC activity was efficiently inhibited by resminostat and the same degree of inhibition was observed in the presence of sorafenib (Supplementary Figure 6).

Overall, here we have demonstrated that HCC cell lines respond to resminostat inducing cell death, which correlated with an apoptotic process. Moreover, a collaborative effect between resminostat and sorafenib is observed, particularly in the mesenchymal HLE and HLF cells that are poorly responsive to sorafenib in terms of apoptosis. Interestingly, the toxic effect of both drugs is lower in untransformed liver cells and no significant collaboration among them is observed (Supplementary Figure 7).

### Resminostat affects the expression of EMT-related genes in HCC cells, which correlates with inhibition in their migratory and invasive properties

In previous studies, we described that a mesenchymal phenotype and the expression of the stem-related gene CD44 confers lack of sensitivity to sorafenib-induced apoptosis in HCC cells [16]. Therefore, next we decided to analyze the effect of resminostat on the mesenchymal and stemness phenotype.

**Figure 5:**
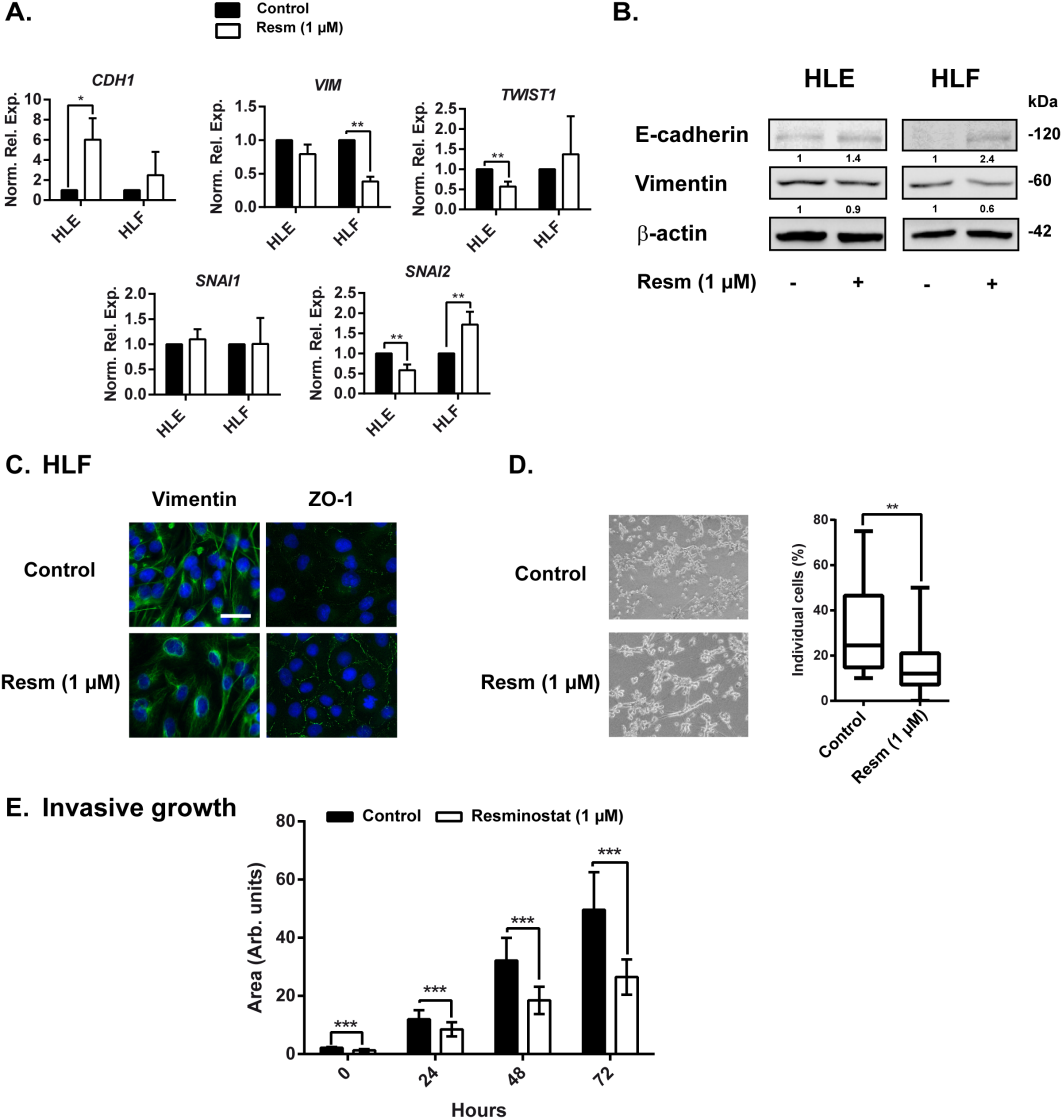
Resminostat affects the expression of EMT related genes **(A)**. HLE and HLF cells were treated with resminostat (1 μM) for 24 h. mRNA expression levels were detected by qRT-PCR, normalized to housekeeping gene *L32* and to respective control (resminostat 0 μM). **(B)**. Western blot analysis of E-cadherin and vimentin in HLF cells after 24 hours of resminostat (1 μM) treatment. β-actin was used as a loading control. Densitometry analysis is below respective bands and normalized to β-actin. **(C)**. HLF cells were treated with resminostat (1 μM) for 24 h. Immunofluorescence analysis of vimentin (green), ZO-1 (green) and DAPI (blue). Scale bar = 25 μm. **(D)**. HLF cells were treated with resminostat (1 μM) for 72 h and seeded on top of a thick collagen I matrix. **Left:** Representative phase contrast images after 24 h (10x objective). **Right:** After 24 h, cells were stained using F-actin and DAPI, visualized by confocal microscopy and the % of individual cells was counted using ImageJ software. The % of individual cells is represented by a box plot with whiskers (min to max). Mann-Whitney t-test was used for statistical analysis. n=20 from a representative experiment. ^**^ p<0.01. **(E)**. HLF cells were treated with resminostat (1 μM) for 72 h and afterwards, the invasive growth in a Collagen I was analyzed. At respective times (0, 24, 48 and 72 h) of the invasion assay, phase contrast images were taken and the area of formed spheroid was analyzed by ImageJ software. ^***^ p<0.001. Mean±SD (n at least 20 spheroids).

Analysis of gene expression after 24 h of resminostat (1 μM) treatment in mesenchymal HLE and HLF cells revealed a shift towards an epithelial phenotype, even though to a different extent in each cell line. In HLE cells, resminostat treatment led to an up-regulation of *CDH1* with a concomitant down-regulation of EMT-inducing transcription factors *TWIST1* and *SNAI2*. In HLF cells we observed a tendency in *CDH1* up-regulation and a significant down-regulation of *VIM* expression without downregulation of *SNAI1* or *SNAI2* (Figure 5A). Western blot analysis revealed a tendency of increased E-cadherin expression and decreased vimentin expression (Figure 5B). Moreover, by immunofluorescence analysis we confirmed vimentin downregulation (Figure 5C). During EMT, cells lose the compact, well-arranged, epithelial structure and gain a spindle-like morphology. During this process, cells lose the expression of ZO-1 protein, among others, that is responsible for tight junctions between cells. Therefore, it was interesting to note that resminostat treatment in HLF cells increased the expression of ZO-1 and re-organised ZO-1 to cell membranes, suggesting a formation of tight junctions and cell clusters, and therefore a more epithelial phenotype. Indeed, when we analyzed the % of individual cells in 3D setting (embedded in collagen I), we could observe a decrease of individual cells and an increase of cells in cell clusters after resminostat treatment (Figure 5D). Similarly, mesenchymal phenotype is connected with higher migration and invasive capacities. We evaluated HLF cells treated with resminostat in an invasive growth assay, in which cells need to invade trough collagen I in a 3D setting. In fact, resminostat treatment decreased their invasive ability (Figure 5E and Supplementary Figure 8).

### Resminostat down-regulates CD44 expression in HCC cells, which reduces their stemness capacity

**Figure 6:**
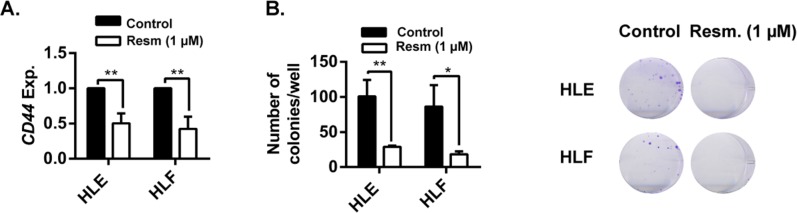
Resminostat decreases *CD44* expression and the stemness capacity of HCC cells **(A)**. HLE and HLF cells were treated with resminostat (1 μM) for 24 h. mRNA expression levels of *CD44* were detected by qRT-PCR, normalized to housekeeping gene *L32* and to respective controls (resminostat 0 μM). Mean±SD (n=3). ^**^ p<0.01. **(B)**. HLE and HLF cells were treated with resminostat (1 μM) for 72 h. 500 cells were seeded in 2 mL of full media (containing 0 and 1 μM resminostat) in a 6-well-plate format. **Left:** Formed colonies were count after 12 days of culture. Mean±SD (n=3). ^*^ p< 0.05, ^**^ p<0.01. **Right:** Representative images of colonies.

Analysis of gene expression after 24 h of resminostat (1 μM) treatment in mesenchymal HLE and HLF cells revealed a downregulation of a stem cell marker related to a mesenchymal phenotype, *CD44*, in both cell lines (Figure 6A). Furthermore, the ability of cells to grow in low dilutions (functional assay of stemness capacity) was significantly decreased by resminostat treatment (Figure 6B).

## DISCUSSION

HCC is one of the most frequent cancers with more than 500,000 new cases observed each year with resistance to conventional chemo- and radiotherapy. Currently, sorafenib is standard of care for patients with advanced HCC, however up to 80% of patients suffer from side effects and even patients who initially respond to sorafenib develop resistance during therapy. The molecular mechanisms for the development of resistance are being extensively studied [17]. Therefore, the development of novel targeted therapy is needed. Emerging evidences suggest that frequent epigenetic alterations in HCC open new possibilities for therapeutic targeting [3, 4]. Although HDAC inhibitors showed antitumor efficacy in preclinical models, there was a lack of efficacy against solid tumors as monotherapy in clinical trials except for some hematological malignancies. HDAC inhibition will be probably most effective as part of combination therapies [18]. Indeed, the efficacy of resminostat, a novel orally available HDAC inhibitor, is being evaluated as a single agent or in combination with sorafenib in HCC [14, 15].

In this study we have demonstrated that resminostat is able to decrease the proliferation and viability in culture of different HCC cell lines in a dose-dependent manner. Mesenchymal HCC cell lines (such as HLE and HLF) were even more sensitive in terms of cell death.

To study the effect of co-treatment with resminostat and sorafenib, we used a low, non-toxic concentration of resminostat (1 μM). HDAC activity was efficiently inhibited by resminostat and this inhibition was maintained in the presence of sorafenib. Furthermore, co-treatment with resminostat and sorafenib had a higher impact on the decrease in p-ERKs levels (relevant targets of sorafenib) than that one exerted by each drug independently. Interestingly, we found a collaborative effect among these drugs, particularly in the mesenchymal HLE and HLF cells, previously identified as unresponsive to sorafenib in terms of apoptosis [16]. Indeed, percentage of hypodiploid cells and PARP cleavage (as hallmark of caspase activation) were higher in the combined treatment. Our results are in agreement with a recent study that demonstrate synergistic effects between resminostat and sorafenib in HCC cells [19]. Furthermore, a previous study using the HDAC inhibitor MPT0E028 also showed synergy with sorafenib *in vitro* and *in vivo* HCC models [20].

The association between EMT and sorafenib resistance, either as a trigger or as a result of treatment, was reported in various studies. A study by Zijl et al. demonstrated that mesenchymal HCC cell lines show higher resistance to cytotoxic drug, including sorafenib [21]. In a study by van Malenstein et al., long-term treatment of HCC cells with sorafenib lead to EMT with concomitant increased invasion, risk of rebound growth and resistance, phenomenon also observed in patients [22]. Previous studies from our group demonstrated that the resistance to sorafenib is associated to a mesenchymal phenotype and expression of mesenchymal stem-related genes, in particular, CD44. Switching to an epithelial phenotype and down-regulation of CD44 sensitized the mesenchymal HCC cells to sorafenib [16].

Recent evidence suggested that cancer cells undergo dynamic and reversible transitions between multiple phenotypic states, ranging from a fully differentiated epithelial state to a dedifferentiated mesenchymal state. Importantly, the fully mesenchymal state is not permanent, as cells might revert to an epithelial state through a mesenchymal-epithelial transition (MET) [23]. The plasticity and reversibility of this process requires not only reprogramming of gene expression, but also epigenetic regulation [24].

Based on demonstrated evidence that the resistance to sorafenib in HCC could be a result of EMT processes and that epigenetics might play a role in EMT induction, we went on to analyse the effect of resminostat in the mesenchymal HLE and HLF cell lines in terms of induction of phenotypical changes. Indeed, gene expression analysis of EMT markers and EMT transcription factors confirmed that resminostat is able to shift cells towards a more epithelial phenotype, even though not to a complete epithelial state and to a different extend in each of the cell line analyzed. Furthermore, a marker of cell-cell junctions, ZO-1, was localized in the cell membranes after resminostat treatment, suggesting induction of an epithelial phenotype. Moreover, the % of individual cells after resminostat treatment was decreased, which suggests the increase in cell-cell junctions. A functional assay of invasive growth in 3D (collagen I) further demonstrated that the shift towards a more epithelial phenotype evidenced in resminostat-treated HLF cells provoked a significant decrease in their invasive capacities. Previous studies also would suggest that epigenetic modulation by HDACs affects the HCC cell phenotype. Indeed, downregulation of HDAC1 by shRNA led to an increase in the levels of ZO-1 and E-cadherin, a decrease in Vimentin and consequent suppression of migration [6]. Moreover, HDAC1 was observed to directly down-regulate ZO-1 and E-cadherin expression in HCC cells, and therefore there might be a possible linkage between HDAC1 overexpresssion and EMT-related invasiveness of HCC [25].

Accumulating evidence in HCC has demonstrated the existence of a small subset of cancer cells with properties of stem cells, such as self-renewal and differentiation. Importantly, the development of cancer recurrence, metastasis and resistance to conventional therapy is often attributed to the presence of cancer stem cells [26]. Increasing evidence suggests that epigenetic mechanisms play a role in the induction of cancer stem cells, and that targeting those mechanisms could induce differentiation and sensitize these cells to therapy [27]. The connection between EMT and stemness in cancer has been widely studied [28]. Indeed, here we are showing that the shift towards an epithelial phenotype by resminostat treatment correlates with downregulation of a cancer stem cell marker, CD44, and with a lower ability to form colonies (a functional assay of stemness capacity). We previously found a cross-talk between the Transforming Growth Factor (TGF)-β pathway and the acquisition of a mesenchymal-like phenotype with up-regulation of CD44 expression in HCC cell lines, CD44 playing an active role in protecting these cells from sorafenib-induced apoptosis [16, 29]. Thus, a mesenchymal profile and high expression of CD44, linked to activation of the TGF-β pathway, may provoke lack of response to sorafenib in HCC patients. Here we propose that the effects of resminostat reversing this phenotype would favor the sensitization of these unresponsive cells to sorafenib-induced cell death.

In conclusion, resminostat prevents growth of HCC cells through the induction of cell death. In mesenchymal HCC cells, which are less sensitive to sorafenib, resminostat potentiates the response to sorafenib-induced apoptosis. We demonstrate for the first time that resminostat induces a switch from a mesenchymal towards an epithelial phenotype and down-regulates CD44 expression, which correlates with lower invasive and stemness properties. This effect could justify the sensitization of mesenchymal HCC cells to sorafenib-induced apoptosis.

## MATERIALS AND METHODS

### Cell culture

Hep3B cells were obtained from the European Collection of Cell Cultures (ECACC). HLE and HLF cells were from the Japanese Collection of Research Bioresources Cell Bank (JCRB Cell Bank). The human liver cell line CCL-13 (Chang liver, CHL) was from the American Type Culture Collection. Cell lines were never used in the laboratory for longer than four months after receipt or resuscitation. Characteristics of the HCC cell lines used are in Supplementary Materials and Methods. They were re-authenticated in 2014 by CLS (Cell Line Service, Eppelheim, Germany). Resminostat and sorafenib were prepared in dimethyl sulfoxide (DMSO) and diluted to working concentration (0-10 μM). Control condition always contained the same amount of DMSO as respective samples. Cells were maintained in DMEM media from Lonza (Basel, Switzerland) supplemented with 10% fetal bovine serum (FBS) from Sera Laboratories International (Cinder Hill, UK, GB), Penicillin (100 U/mL), Streptomycin (100 μg/mL), Amphotericin (2.5 μg/mL) and L-glutamine (2 mM), and were maintained in a humidified atmosphere at 37°C, 5% CO_2_. Cells were observed under an Olympus 70iX microscope and photographed with a Spot 4.3 digital camera. When cells were cultured on thick layers of collagen I, fibrillar bovine dermal collagen I (no. 5005; PureCol, Advanced BioMatrix) was prepared at 1.7 mg/mL in DMEM according to the manufacturer's protocol. After collagen I polymerization (4 h at 37°C, 10% CO_2_) cells were seeded on top of the collagen in medium containing 10% FBS and allowed to adhere for 24 h.

### Cell proliferation assay

Cells were seeded in a 24-well plate (15 000 cells/well) in 500 μL of full DMEM supplemented with 10% FBS. After 24 h, the media was replaced with media containing respective compounds. At specific time points (0, 24, 48 and 72 h), cells were washed with PBS and stained with crystal violet (0.2% crystal violet in 2% ethanol solution) for 30 min and later washed with distilled water and dissolved in 10% SDS on a shaker for 30 min. The absorbance was analyzed on a plate reader at 560 nm.

### Analysis of cell death

After respective treatments, media (including floating cells) and remaining cells were collected and centrifuge at 1,200 rpm for 5 min at room temperature. The cell pellet was resuspended in 200 μL of PBS. Propidium Iodide (PI) at a final concentration of 1 μg/ml was added to the samples and immediately analyzed by flow cytometry (Gallios flow cytometer by Beckman-Coulter). Results were analyzed with Beckman Coulter's Kaluza flow cytometry analysis software (version 1.1).

### Flow cytometric analysis of cell cycle and apoptosis

After respective treatments, media (including any floating cells) and all remaining cells were collected. 2×10^5^ cells/sample were fixed with 70% ice-cold ethanol for 2 min while vortexing. Samples were centrifuged at 2,500 rpm for 5 min at 4°C. The cell pellet was resuspended in 200 μL of PBS, RNAse A at a final concentration of 30 ng/mL (Sigma-Aldrich) was added and samples were cultivated for 30 min at 37°C. PI at a final concentration of 1 μg/mL was added to the samples 15 min prior analysis by flow cytometry (Beckman-Coulter). Results were analyzed with Beckman Coulter's Kaluza flow cytometry analysis software (version 1.1).

### Colony formation at low cell densities

After respective treatments, 500 cells were seeded in each well of a 6-well dish in triplicates in 2 mL of full media and were allowed to grow into colonies. After 12 days, colonies were stained with a solution of crystal violet (0.2% in 2% ethanol) and counted using ImageJ software and are represented as a number of colonies formed per well.

### Invasive growth assays

Cells were resuspended in low viscosity media containing respective treatments (detailed in figure legend) in a concentration of 4×10^5^ cells/mL and suspended as hanging drops for 72 h at 37°C (5% CO_2_), to cluster into spheroids. Then, spheroids were resuspended in a solution of collagen I (1.7 mg/mL in DMEM, containing respective treatments) and incubated at 37°C (10% CO_2_). Phase contrast pictures were taken at 0, 24, 48 and 72 h. For quantification, the area occupied by spheroids was calculated using ImageJ software.

### Immunofluorescence studies in 2D

Fluorescence microscopy studies were performed as described previously [30]. Additional information and list of used antibodies in Supplementary Materials and Methods. Cells were visualized with a Nikon eclipse 80i microscope. Representative images were taken with a Nikon DS-Ri1 digital camera and edited in Adobe Photoshop.

### Immunofluorescence in cells cultured on thick layers of collagen I

Immunostaining of cells seeded on top of a thick collagen I matrix was performed as described previously [31]. Additional information and list of used antibodies in Supplementary Materials and Methods. For the imaging, collagen gels with immunostained cells were transferred to glass-bottomed dishes and visualized on a Zeiss LSM 510 Meta confocal microscope (Carl Zeiss) with C-Apochromat 40x / 1.2 NA (water) and Zen software (Carl Zeiss). Confocal Z-slice images were analysed using ImageJ.

### Western blot analysis

Procedure was carried out as described previously [30]. Additional information and list of used antibodies in Supplementary Materials and Methods.

### Analysis of histone deacetylase activity

HDAC activity was analyzed by a fluorometric assay kit (Abcam, ab156064) according to manufacturer's instructions.

### Analysis of gene expression

RNeasy Mini Kit (Qiagen, Valencia, CA) was used for total RNA isolation. Reverse transcription was done using the High Capacity Reverse Transcriptase kit (Applied Biosystems, Foster City, CA, USA), and 1 μg of total RNA from each sample for complementary DNA synthesis. For qRT-PCR, expression levels were determined in duplicate in a LightCycler 480 Real-time PCR system, using the LightCycler 480 SYBR Green I Master Mix (Roche Diagnostics GmbH, Mannheim, Germany). Human specific primers designed by Integrated DNA Technologies (IDT) are listed in Supplementary Materials and Methods.

### Statistical analyses

All data represent at least three experiments and are expressed as the mean ± standard deviation (SD). Differences between groups were compared using either Student's t-test assuming parametric distribution and by Mann-Whitney t-test assuming nonparametric distribution. Statistical significance was assumed when p<0.05. In all cases statistical calculation was performed using GraphPad Prizm software (Graph-Pad for Science Inc., San Diego, CA, USA).

## SUPPLEMENTARY MATERIALS FIGURES



## Abbreviations

HCC: human hepatocellular carninoma
EMT: epithelial-mesenchymal transition
HDAC: histone deacetylase
ZFP64: zing finger protein 64
FBS: fetal bovine serum
DMSO: dimethyl sulfoxide
PI: propidium iodide
PARP: poly ADP ribose polymerase
ZO-1: zonula occludens-1
CDH1: E-cadherin
VIM: vimentin
MET: mesenchymal-epithelial transition
EMT: epithelial-mesenchymal transition
SD: standard deviation.
