# Supplementary material for: Resminostat induces changes in epithelial plasticity of hepatocellular carcinoma cells and sensitizes them to sorafenib-induced apoptosis
Source: Oncotarget. 2017 Nov 30;8(66):110367–79. doi: 10.18632/oncotarget.22775 (PMC5746389; doi:10.18632/oncotarget.22775)
Supplement: Supplementary file 1 [file oncotarget-08-110367-s001.pdf]

Resminostat induces changes in epithelial plasticity of hepatocellular carcinoma cells and sensitizes them to sorafenib-induced apoptosis

SUPPLEMENTARY MATERIALS

Characteristics of human HCC cell lines used in the study

Cell line	Tumor type	Morphology	Differentiation grade	*TP53* status	HBV	Other characteristics
Hep3B	Human negroid hepatocarcinoma	Epithelial	Well differentiated	Deleted	Integrated	Deficient in functional RB1; mutations within *FAS*
HLE	Human hepatoma (non differentiated)	Diffusely spreading cells	Poorly differentiated	Mutated G244A R249S V272M	Negative	-
HLF	Human liver hepatocarcinoma	Diffusely spreading cells	Poorly differentiated	Mutated p.G244A	Negative	-

Immunofluorescence studies in 2d

For F-actin, cells were fixed with 4% paraformaldehyde (PFA) in PBS for 30 min at room temperature, incubated with a blocking solution (1% BSA, 10% FBS) before addition of the primary antibody (1:500 dilution, 1 h, room temperature). For VIMENTIN, E-CADHERIN and ZO-1, cells were fixed with methanol (100%; −20°C, 2 min), incubated with blocking solution and cultivated with primary antibodies diluted in 1% BSA (1:50 dilution, 1 h, room temperature). After primary antibody incubation, cells were washed several times with PBS, incubated with Alexa Fluor 488-conjugated anti-rabbit or Alexa Fluor 488-conjugated anti-mouse (1:1000 dilution, 1 h, room temperature) and mounted with Vectashield mounting medium. Cells were visualized with a Nikon eclipse 80i microscope. Representative images were taken with a Nikon DS-Ri1 digital camera and edited in Adobe Photoshop.

Immunofluorescence in cells cultured on thick layers of collagen i

Cells were fixed during 15 min at room temperature by adding 50 µL of PFA 12% to cells seeded on 100 µL of matrix (final concentration of PFA is 4% considering collagen matrix volume) and washed 3 times with PBS during 5 minutes. Afterwards, cells were permeabilized during 10 min using a solution containing 0.2% Triton X-100 in PBS-BSA 5% and washed again 3 times with PBS during 5 min. Next, samples were blocked in a PBS-BSA 5% solution during 20 min. For F-actin cells were stained during 1 h at room temperature with Alexa Fluor 546-phalloidin probe diluted 1:500 in blocking solution, and washed again 5 times with PBS during 5 min. During the second last wash nuclei were stained with a DAPI solution 1:200.

Western blot analysis

Total protein extracts were obtained using a lysis buffer containing 30 mM Tris–HCl pH 7.5, 5 mM EDTA, 150 mM NaCl, 1% Triton X-100, 0.5% sodium deoxycholate, 0.1% SDS and 10% glycerol (1 h at 4°C; centrifugation at 13,000 rpm, 10 min, 4°C). Protein concentration was measured with the BCA Protein Assay kit (Pierce). β-ACTIN is shown as a loading control. Antibodies were used at a 1:1000 dilution, except for β-ACTIN (1:3000). Images were processed with Adobe Photoshop CS5.

List of primers

Real-time quantitative PCR reactions were performed using the following human specific primers designed by Integrated DNA Technologies (IDT):

L32 Forward: 5’-AACGTCAAGGAGCTGGAAG-3’

Reverse: 5’-GGGTTGGTGACTCTGATGG-3’

CDH1 Forward: 5’-CCCAATACATCTCCCTTCACAG-3’

List of antibodies used in the study

Primary antibody	Secondary antibody	Company	Reference code	Application
E-CADHERIN	Anti-mouse	BD Biosciences	BD-610182	IF, WB
VIMENTIN	Anti-mouse	Sigma-Aldrich	V6630	IF, WB
ZO-1	Anti-rabbit	Invitrogen	61-7300	IF
F-ACTIN	TRITC-conjugated	Sigma-Aldrich	P1951	IF
β-ACTIN	Anti-mouse	Sigma-Aldrich	A5441	WB
PARP	Anti-rabbit	Cell Signaling	9542	WB
phospho-p42/44 MAPK	Anti-rabbit	Cell Signaling	9101	WB
**Secondary antibody**	**Company**	**Reference code**	**Application**	
Alexa 488 goat anti mouse	Life Technologies	A11001	IF	
Alexa 488 goat anti rabbit	Life Technologies	A11008	IF	
Anti-mouse	GE Healthcare	NA931V	WB	
Anti-rabbit	GE Healthcare	NA934V	WB	

IF: Immunofluorescence; WB: Western Blot;

Reverse 5’-CCACCTCTAAGGCCATCTTTG-3’

VIM Forward: 5’- GGAAGCCTAACTACAGCGAG -3’

Reverse: 5’- CAGAGTCCCAGATGAGCATTG -3’

SNAI1 Forward: 5′-GCTGCAGGACTCTAATCCAGAGTT-3′

Reverse: 5’-GACAGAGTCCCAGATGAGCATTG-3’

SNAI2 Forward: 5′-ACACATTAGAACTCACACGGG -3′

Reverse: 5’- TGGAGAAGGTTTTGGAGCAG -3’

ZEB1 Forward: 5′-ACCCTTGAAAGTGATCCAGC-3′

Reverse: 5’-CATTCCATTTTCTGTCTTCCGC-3’

TWIST1 Forward: 5′-CTCAGCTACGCCTTCTCG-3′

Reverse: 5’-ACTGTCCATTTTCTCCTTCTCTG-3’

EPCAM Forward: 5′-CAATGCAGGGTCTAAAAGCTG -3′

Reverse: 5′-CACCCATCTCCTTTATCTCAGC-3′

CD133 Forward: 5′-GTGGATGCAGAACTTGACAAC-3′

Reverse: 5′-ACCCTTTTGATACCTGCTACG-3′

CD44 Forward: 5′-TGGGTGTGTCCTTCGCTCGC-3′

Reverse: 5′-GCGGACCGAACCTGGCAGAG-3′

CD90 Forward: 5′-GAGATCCCAGAACCATGAACC-3′

Reverse: 5′-TGCTGGTATTCTCATGGCG-3′

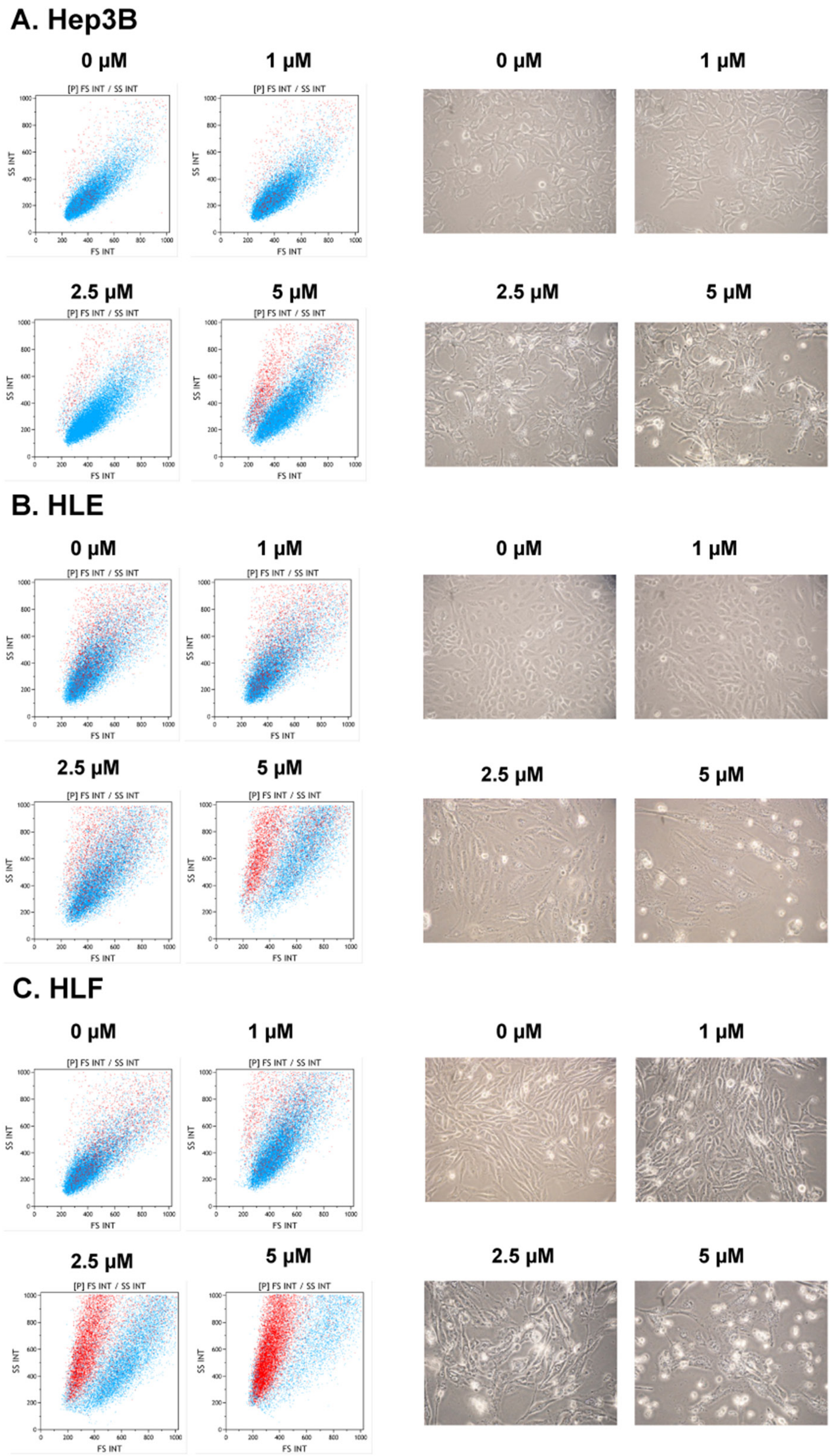

Supplementary Figure 1: Resminostat induces cell death in HCC cells. Hep3B **(A)**, HLE **(B)** and HLF **(C)** were treated with resminostat (0-5 µM) for 72 h. Representative images of flow cytometry analysis of non-viable (PI-positive-red), viable cells (PI-negative-blue) and phase contrast microscopy images are shown.

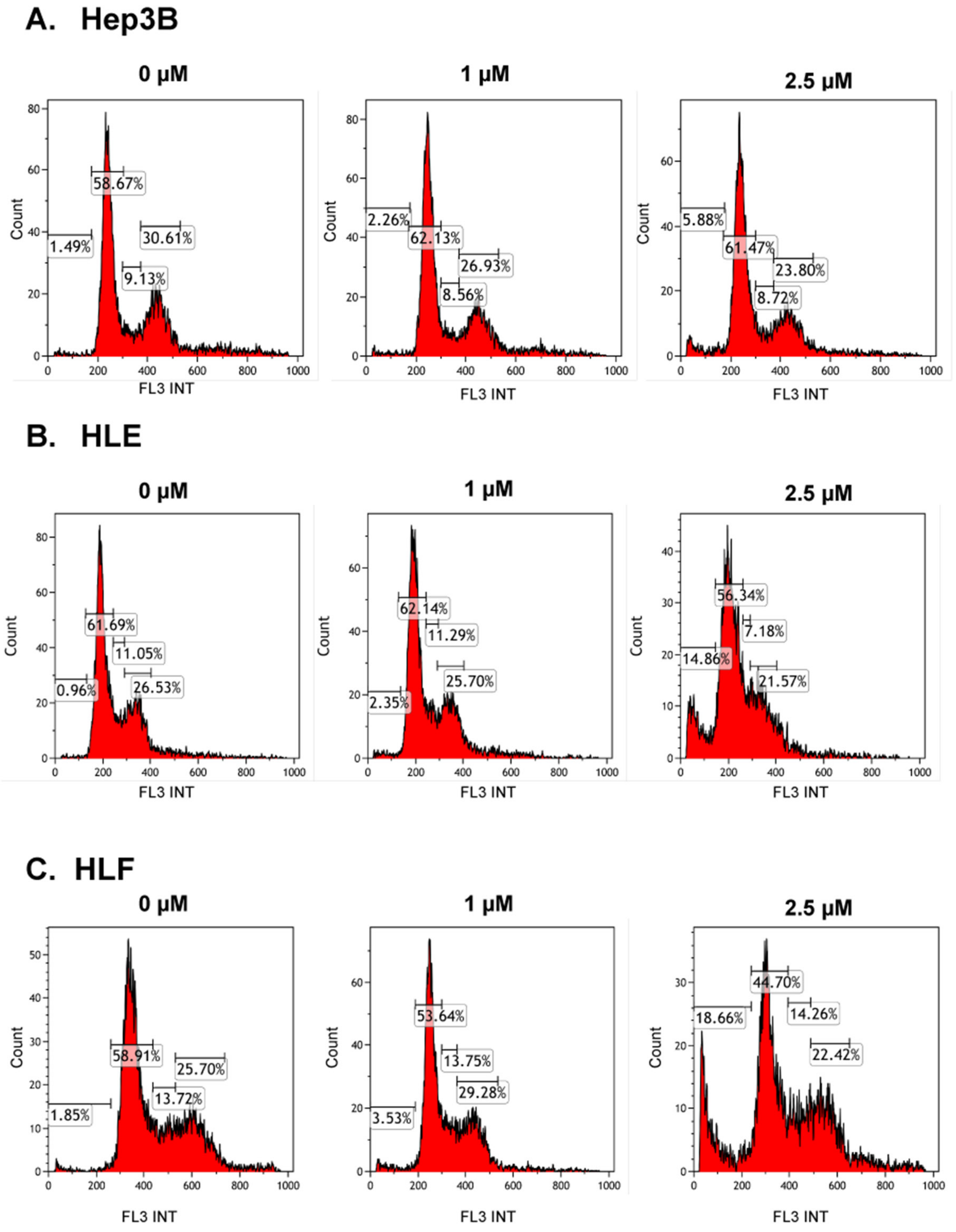

Supplementary Figure 2: Resminostat induces apoptosis in HCC cells. Hep3B **(A)**, HLE **(B)** and HLF **(C)** were treated with resminostat (0-2.5 μM) for 72 h. Representative plots of cell cycle analysis are shown.

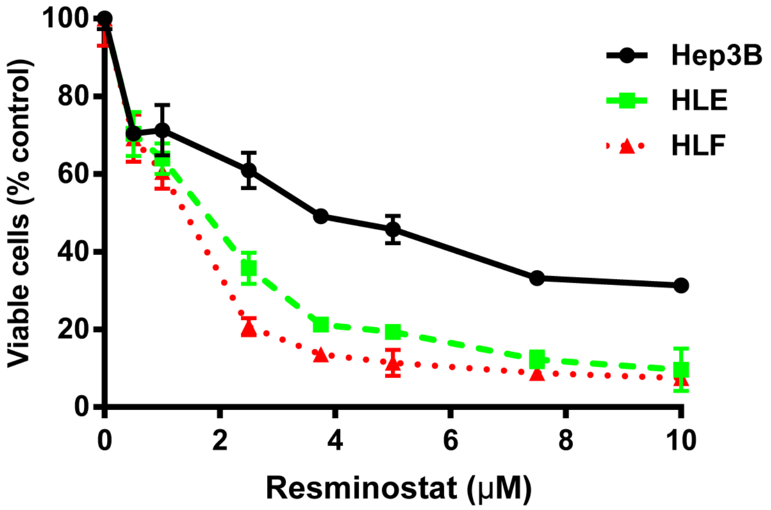

Supplementary Figure 3: Determination of IC50 of resminostat in combination with sorafenib (5 µM). Hep3B, HLE and HLF cells were treated with sorafenib (5 µM) and resminostat in a range of concentrations (0-10 µM). Viable cells were analyzed by crystal violet staining after 72 h of respective treatments and normalized to control. Mean ±SD (n=3).

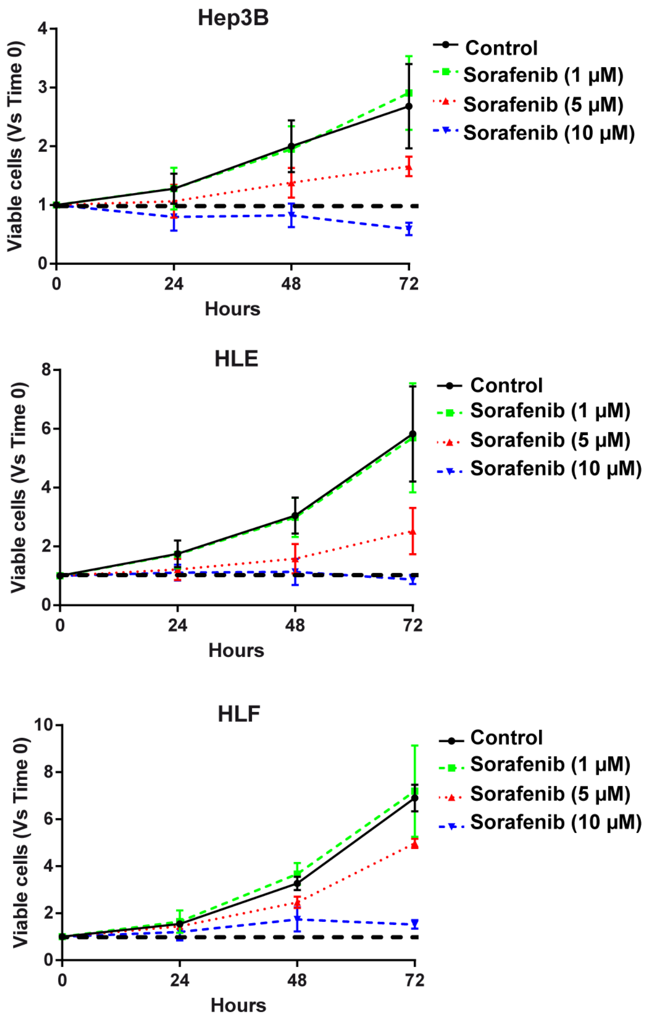

Supplementary Figure 4: Sorafenib displays cytotoxic effect only in the epithelial Hep3B cells. Hep3B, HLE and HLF cells were treated with sorafenib in a range of concentrations (0-10 µM). Viable cells were analyzed by crystal violet staining after 24, 48 and 72 h of respective treatments and are represented as viable cells versus time 0. Mean ±SD (n=3).

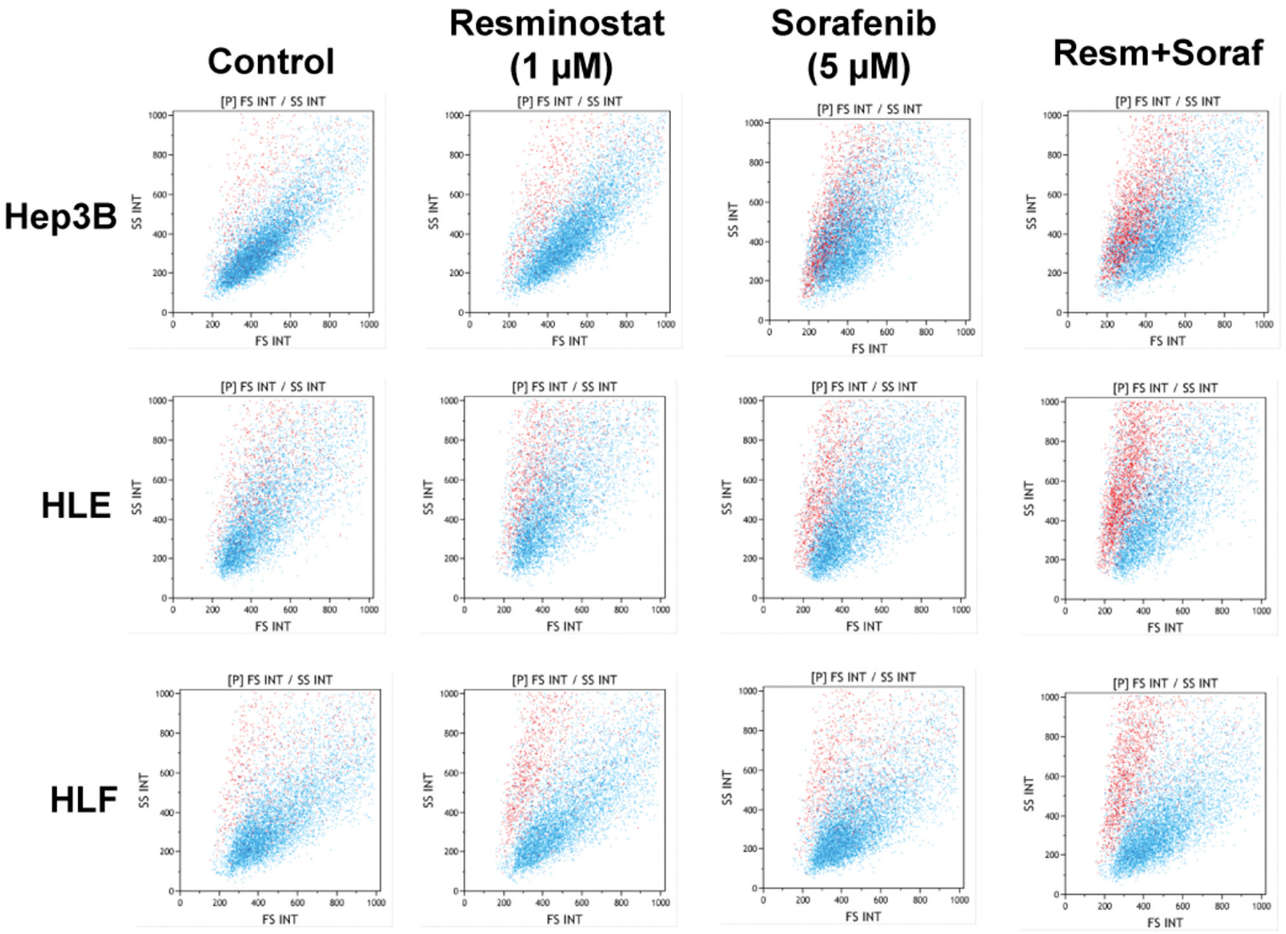

Supplementary Figure 5: Effect of combined treatment of resminostat and sorafenib. Hep3B, HLE and HLF cells were treated with resminostat (0 µM), resminostat (1 µM), sorafenib (5 µM) or a combination of resminostat (1 µM) and sorafenib (5 µM) for 72 h. The % of non-viable cells (PI-positive) was determined by flow cytometry. Representative plots are shown.

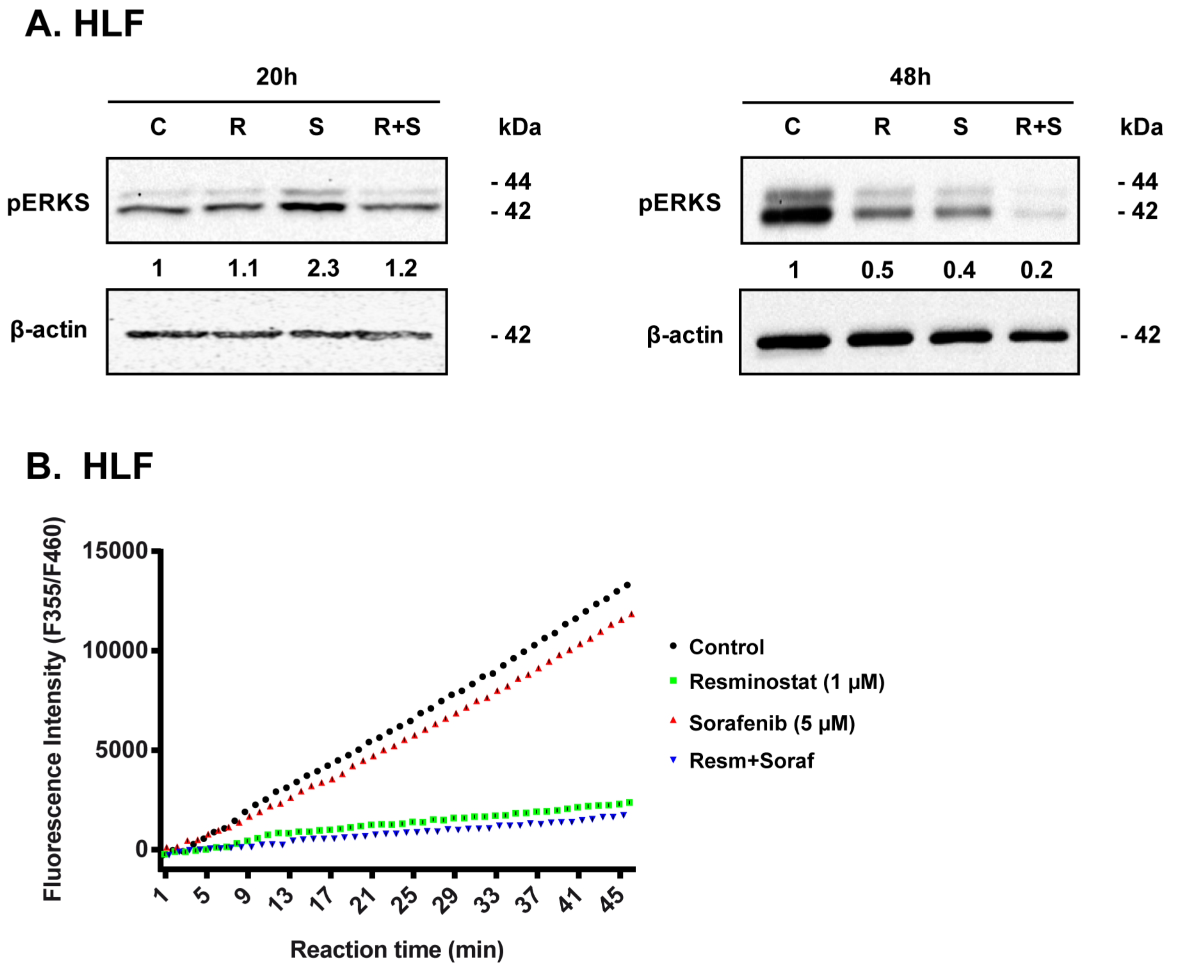

Supplementary Figure 6: The effect of resminostat and sorafenib treatment on pERKs and HDAC activity. HLF cells were treated with resminostat (0 µM), resminostat (1 µM), sorafenib (5 µM) or a combination of resminostat (1 µM) and sorafenib (5 µM). **(A)** Western blot analysis of pERKs in HLF cells after 20 h and 48 h or respective treatments. β-actin was used as a loading control. Densitometry analysis is shown below respective bands and is normalized to β-actin. **(B)** HDAC activity was detected by a commercial kit after 24 h of respective treatments and is represented as Fluorescence intensity (F355/F460) over time (min). A representative experiment is shown (n=3).

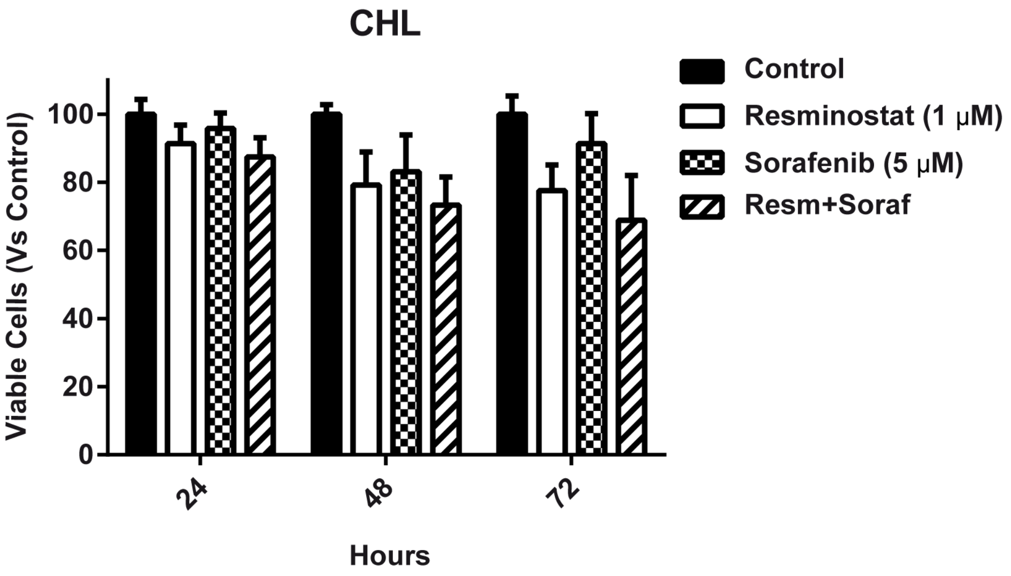

Supplementary Figure 7: Response of a human liver cell line CCL-13 (Chang liver, CHL) to resminostat, sorafenib or the combination of both drugs. CHL cells were treated with resminostat (1 µM), sorafenib (5 µM) or a combination of resminostat (1 µM) and sorafenib (5 µM) at respective times. Viable cells were analyzed by crystal violet staining and normalized to control. Mean ±SD (n=3).

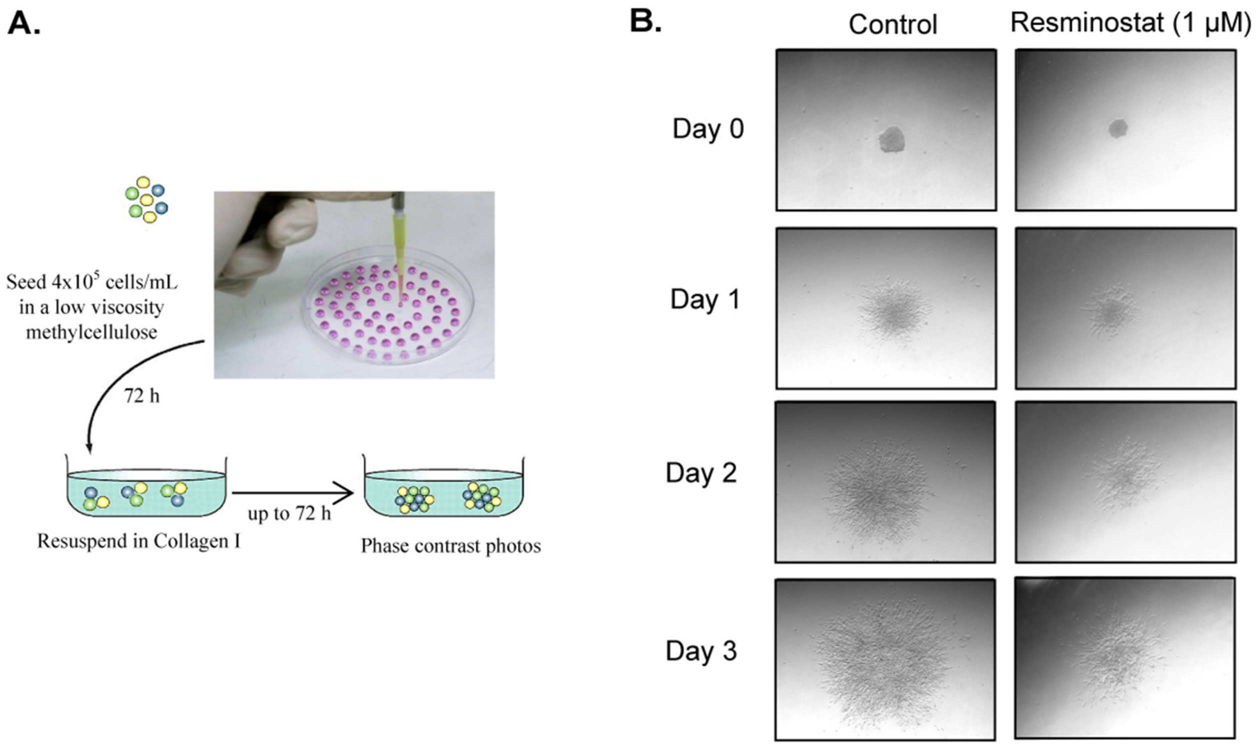

Supplementary Figure 8: Resminostat treatment decreases the invasive growth ability in HLF cells. HLF cells were treated with resminostat (1 µM) for 72 h. The methodological approach for invasive growth assay is shown **(A)**. Representative phase contrast images at respective time points are shown at magnification 4x **(B)**.
